# Temperature and water availability drive insect seasonality across a temperate and a tropical region

**DOI:** 10.1098/rspb.2024.0090

**Published:** 2024-06-19

**Authors:** Laura J. A. van Dijk, Brian L. Fisher, Andreia Miraldo, Robert M. Goodsell, Elzbieta Iwaszkiewicz-Eggebrecht, Dimby Raharinjanahary, Eric Tsiriniaina Rajoelison, Piotr Łukasik, Anders F. Andersson, Fredrik Ronquist, Tomas Roslin, Ayco J. M. Tack

**Affiliations:** ^1^ Department of Bioinformatics and Genetics, Swedish Museum of Natural History, Stockholm 114 18, Sweden; ^2^ Entomology, California Academy of Sciences, San Francisco, CA 94118, USA; ^3^ Madagascar Biodiversity Center, Parc Botanique et Zoologique de Tsimbazaza, Antananarivo 101, Madagascar; ^4^ Institute of Environmental Sciences, Faculty of Biology, Jagiellonian University, 30-387 Kraków, Poland; ^5^ Science for Life Laboratory, Department of Gene Technology, KTH Royal Institute of Technology, Stockholm 171 21, Sweden; ^6^ Department of Ecology, Swedish University of Agricultural Sciences, 750 07 Uppsala, Sweden; ^7^ Department of Ecology, Environment and Plant Sciences, Stockholm University, 114 19 Stockholm, Sweden

**Keywords:** arthropods, flying insect biomass, phenology, seasonality, spatial distribution, tropical and temperate climates

## Abstract

The more insects there are, the more food there is for insectivores and the higher the likelihood for insect-associated ecosystem services. Yet, we lack insights into the drivers of insect biomass over space and seasons, for both tropical and temperate zones. We used 245 Malaise traps, managed by 191 volunteers and park guards, to characterize year-round flying insect biomass in a temperate (Sweden) and a tropical (Madagascar) country. Surprisingly, we found that local insect biomass was similar across zones. In Sweden, local insect biomass increased with accumulated heat and varied across habitats, while biomass in Madagascar was unrelated to the environmental predictors measured. Drivers behind seasonality partly converged: In both countries, the seasonality of insect biomass differed between warmer and colder sites, and wetter and drier sites. In Sweden, short-term deviations from expected season-specific biomass were explained by week-to-week fluctuations in accumulated heat, rainfall and soil moisture, whereas in Madagascar, weeks with higher soil moisture had higher insect biomass. Overall, our study identifies key drivers of the seasonal distribution of flying insect biomass in a temperate and a tropical climate. This knowledge is key to understanding the spatial and seasonal availability of insects—as well as predicting future scenarios of insect biomass change.

## 1. Introduction

Insects play a key role in natural as well as agricultural systems by providing ecosystem functions such as pollination and decomposition, and by offering a food source for a variety of organisms [1–3]. The quantity of food and ecosystem functions provided varies with the amount of active insect biomass in an ecosystem, which fluctuates through space and time [4,5]. While studies have described trends of insect biomass across years for various regions [6–8], we lack insights into the seasonal dynamics of insect biomass across large spatial scales, as well as into the drivers of these spatial and seasonal distributions. Since all trophic layers directly or indirectly depend on insects, the timing and availability of insects throughout the year affects entire ecosystems, and understanding their drivers is thus a priority [9,10].

Climatic conditions are important drivers of the spatial distribution of insect biomass [11,12]. The temperature of the environment determines the rates of insect development, reproduction and activity and positively contributes to the available energy in the system [13]. Therefore, warmer regions are generally expected to sustain larger insect populations compared with colder regions [14], though temperatures above the thermal maxima of insects will have negative effects [15]. Insect clades with a narrower thermal tolerance—like tropical insects adapted to relatively stable temperature regimes—will be more sensitive to changes in temperature [16,17]. Water availability can also affect insect abundances, either directly by increased risk of desiccation during droughts, or indirectly by reducing the availability of nectar, foliage or pollen or by promoting the spread of fungal pathogens across insects [18–20]. Conversely, extreme rainfall will limit periods of insect flight, and could increase insect mortality [21,22]. In tropical climates, temperatures are more constant throughout the year, and unlikely to fall below the thermal minima of insects [23]. Thus, water availability is likely a more important driver of spatial variation in insect abundances in the tropics [24,25].

Besides climate, different habitat types can cause spatial heterogeneity of flying insect biomass, and human-induced alterations of landscapes could have a large influence on the occurrences and abundances of insects [11,26]. Studies report less flying insect biomass in urban areas [27], but higher biomass in non-irrigated agricultural lands, pastures and orchards compared with more natural landscapes such as forests. Such contrasts may be partly due to increased insect movement in more open landscapes [12]. Studies conducted at larger spatial scales are scarcer in tropical regions [28–30], and mostly focus on specific taxa rather than entire insect faunas [31,32]. Overall, we lack insights into general drivers behind insect biomass distributions in the tropics. Hence, to identify general patterns and drivers of the spatial distribution of insect biomass, we need large-scale sampling efforts across multiple countries, including both temperate and tropical climates [33].

Seasonality (i.e. within-year temporal dynamics) determines the quantity of flying insect biomass through time. The seasonality of insects may vary across climatic gradients; that is, regions may differ in terms of their seasonal amplitude (i.e. difference between highest and lowest insect biomass), and in the timing and speed of increases and decreases in insect abundances. Since insects are ectotherms, accumulated heat may explain spatial differences in their seasonality in temperate zones, with earlier growing seasons and higher peaks in species abundances in warmer compared with colder regions [34,35]. Another driver of specific importance to the temperate zone is snow cover. Since most insects will spend their diapause phase in or close to the soil, snow may determine overwintering conditions. It can isolate the soil from extremely cold temperatures, protect against direct exposure to solar radiation, and contribute to soil moisture once the snow melts [36–38]. Periods of seasonal activity tend to be longer in tropical than in temperate climates, with less pronounced peaks in species abundances [5]. Still, seasonality occurs in the tropics, too, where insect biomass tends to be highest during the rainy season [5]. How local communities respond to the dry and wet season can also vary with the landscape. In dry forests, insect biomass may increase faster at the start of the rainy season than in rainforests [39]. Moreover, insect abundances may decrease more during the dry season in hotter and drier regions than in cooler regions, owing to accentuated desiccation risks [24]. Conversely, hot regions may sustain higher insect abundance during the rainy season, when accumulated heat promotes insect activity without desiccation risks [5]. While seasonality has been studied for certain groups of insects, like butterflies and beetles [40–42], we lack a more comprehensive understanding of trends in insect seasonality in relation to climatic and environmental gradients across temperate and tropical zones [43]. In particular, we need large-scale studies across zones conducted throughout the year.

While seasonality trends might explain much variation in flying insect biomass throughout the year, short-term fluctuations in weather conditions could add variation at a daily or weekly scale. For example, insect activities could increase or decrease with day-specific temperature [44,45] or drop during rainy periods [46]. During periods of drought, lowered water availability could increase insect or host plant desiccation and decrease activity [19]. Identification of such short-term drivers will further refine our understanding of the seasonal availability of insects. Nonetheless, compared with the effects of monthly or yearly climatic conditions [5,12,47,48], the effects of short-term weather fluctuations on flying insect biomass remain poorly explored [40].

We aimed to describe the spatial distribution of overall, yearly flying insect biomass, as well as the seasonality of flying insect biomass. To uncover general drivers behind spatial and seasonal patterns, we combine a massive survey of the entire insect fauna of a temperate and a tropical country, Sweden and Madagascar. Specifically, our research questions targeted both regional patterns within Madagascar and Sweden, and a comparison between the two climatic zones:

1. How is flying insect biomass distributed through space?
  a. What are the climatic drivers (accumulated heat, snow depth, rainfall, soil moisture) behind the spatial distribution in biomass?
  b. Is any residual variation in the spatial distribution of flying insect biomass explained by landscape characteristics (habitat type and vegetation cover)?
2. How is flying insect biomass distributed through time?
  a. What are the drivers behind spatial variation in insect seasonality, in terms of local climate and landscape? That is, does insect seasonality differ between warmer and colder sites, snowier and less snowy sites, wetter and drier sites, more vegetated and less vegetated sites, and among habitat types?
  b. Can short-term variations in weather conditions, including accumulated heat, rainfall and soil moisture, explain residual variation in flying insect biomass across seasons?

For our *a priori* expectations related to these questions, see electronic supplementary material, table S1. By intensively sampling a tropical and a temperate country through space and time, our study provides a first and unique insight into the spatiotemporal dynamics of insects in tropical and temperate zones.

## 2. Material and methods

### (a) Study area and data collection

We sampled insects during one year across a temperate (Sweden; 450 000 km^2^; latitude 55.3° to 69.1°) and a tropical (Madagascar; 590 000 km^2^; latitude −25.6° to −12.0°) country (electronic supplementary material, figure S1). The climate in Sweden ranges from oceanic to sub-Arctic, in Madagascar from tropical humid to dry tropical.

Insects were collected with Malaise traps and preserved in 95% ethanol. Site selection in Sweden followed a stratified design based on the major habitat types: forests, grasslands, croplands, alpine and urban areas [49] (electronic supplementary material, figure S1). The proportion of Malaise traps placed in each habitat was decided based on the approximate area covered by each of these habitat types in Sweden. To ensure sufficient replication for each of the habitat types, sampling in rarer habitats was up-weighted (for croplands and wetlands, each covering 8% of area in Sweden, and grasslands and urban areas, each covering 3% of area in Sweden), while sampling in forest sites (covering 60% of area in Sweden) was down-weighted. In Madagascar, traps were placed in both rainforests and dry forests within protected areas (electronic supplementary material, figure S1). Rather than selecting hot-spots of particularly high insect activity, we immersed the traps within the habitats of interest (for details, see supplementary electronic supplementary material, text S1)—an approach that enabled comparisons among sites. In Sweden, we ran 195 Malaise traps from January to December 2019; in Madagascar, we ran 50 Malaise traps from August 2019 to July 2020. Samples were collected and weighed at weekly to biweekly intervals (electronic supplementary material, text S1), with the frequency of sample collection in Sweden adjusted to the season. Within seasons, all samples were collected with the same intervals. By this approach, we avoided any saturation of samples, while keeping sampling times standardized between traps. After collection, none of the samples was filled to more than half of its volume with insects. Methods for wet-weighing of biomass were optimized as described in [50]. Samples were drained of ethanol and weighed to the nearest 0.001 g. For interpolation of missing values, see electronic supplementary material, text S2. Total flying insect biomass per trap—henceforth ‘total biomass’—was obtained by summing the biomass of all samples taken at each trap, then dividing this sum by the total number of sampling days per trap, hence deriving the flying insect biomass per trap per day (electronic supplementary material, table S2 and text S2).

### (b) Climate and landscape variables

#### (i) Climate variables

To characterize the local climate, we extracted climate data from the ERA5-Land database (ECMWF, Copernicus) [51]. The data extracted included temperature, precipitation and soil moisture for all sites in Sweden and Madagascar, and snow depth for sites in Sweden (for details, see electronic supplementary material, text S3 and figure S2). From hourly temperature data, we calculated day- and site-specific growing degree days (GDD5; henceforth ‘accumulated heat’) as:

$$\mathrm{GDD}5=\;\frac{\mathrm{maximum}\;\mathrm{daily}\;\mathrm{temperature}+\mathrm{minimum}\;\mathrm{daily}\;\;\mathrm{temperature}}{2}-5.$$

Here, we adopted the base value of 5 for calculating growing degree days, as insect activity and growth have been shown to dramatically slow down below 5°C [52–56].

#### (ii) Landscape variables

In Sweden, we distributed Malaise traps among forest, grassland, cropland, wetland, urban areas and alpine areas (as defined by Ståhl *et al*. [49]) in rough proportion to the national extent of these habitats (see 'Study area and data collection' (§2a)). In Madagascar, traps were placed in the two major forest types: tropical dry forests and tropical rainforests. As further characterizations of habitat, we collected data on vegetation cover from the ERA5-Land database (for details, see electronic supplementary material, text S3). Since all traps in Madagascar were situated in forests, we collected additional data on forest cover (within 1 km^2^ from the trap) from the ERA5-Land database, and percentage canopy cover above each trap (for details, see electronic supplementary material, text S3).

### (c) Statistical analyses

Statistical analyses were conducted in R v.4.2.0 [57]. We used the package *lme4* to fit linear mixed models [58], and the *Anova* function in the *car* package to assess significance of the models [59]. Model assumptions were evaluated using the *sjPlot* package [60].

#### (i) Patterns and drivers of spatial distribution of biomass

To relate the spatial distribution of flying insect biomass to regional climate, we modelled total biomass as a function of accumulated heat, rainfall and soil moisture for both Sweden and Madagascar, using linear models (electronic supplementary material, table S2). For Sweden, where all sites receive some snowfall during winter, we added snow depth as an additional predictor. To select a model that best described spatial drivers, we used forward-selection which allowed us to add variables in the following order: accumulated heat, snow depth (only in Sweden), rainfall and soil moisture. The order of addition reflected the *a priori* perceived importance of these drivers, with accumulated heat capturing the overall constraint of energy available to ectotherms, snow depth reflecting the insulative layer for diapausing insects, as well as moisture available to insects upon snow melt, rainfall reflecting overall water availability, and soil moisture reflecting water availability in the soil (electronic supplementary material, table S1). Since the impact of accumulated heat may differ depending on the amount of rainfall, we also included the interaction between accumulated heat and rainfall. Added variables were retained in the model if the Akaike information criterion (AIC) of the model with the new variable included was markedly lower than the AIC of the previously fitted model (ΔAIC≥2). For the terms selected in the final models, see electronic supplementary material, table S2. To investigate whether differences in total flying insect biomass between Sweden and Madagascar were caused by the different habitats sampled in each region, we also fitted the spatial model (as described above) with data from forests only for both Sweden and Madagascar.

To investigate whether unexplained variation in biomass could be due to differences in local landscapes, we modelled the residuals as a function of landscape characteristics. For Sweden, we explored the effect of habitat type, i.e. grassland, cropland, forest, wetland, urban and alpine, and of vegetation cover. For Madagascar, we explored the effect of habitat type (dry versus rain forest), as well as the effect of vegetation cover, percentage forest cover and canopy cover.

#### (ii) Patterns and drivers of temporal distribution of biomass

To relate insect seasonality to climatic and landscape predictors, we modelled biomass per day per trap as a function of ‘seasonality’ with the periodic functions sin(2πd/365) and cos(2πd/365), where *d* is the Julian day of the year. To explore whether seasonality is affected by environmental predictors, we added interactions between seasonality and all environmental variables to the model. Again, we used forward-selection with the retention criteria above, adding terms in the order of the annual models: accumulated heat and its interaction with seasonality, snow depth and its interaction with seasonality (only in Sweden), rainfall and its interaction with seasonality, soil moisture and its interaction with seasonality, vegetation cover and its interaction with seasonality and habitat type and its interaction with seasonality (electronic supplementary material, table S2). To account for repeated sampling, trap ID was included as a random effect. For the terms selected in the final models, see electronic supplementary material, table S2.

#### (iii) Drivers of short-term deviations in biomass

To investigate whether week-to-week fluctuations in weather explain the variation in insect biomass that cannot be attributed to seasonal trends, we modelled the residuals of the temporal models above as a function of weekly weather conditions (i.e. weekly averages of accumulated heat, rainfall and soil moisture, from the week preceding sample collection; electronic supplementary material, table S2), with trap ID added as a random effect.

## 3. Results

### (a) Spatial variation in climate and flying insect biomass in Sweden and Madagascar

Madagascar was characterized by a higher annual mean of accumulated heat, more annual rainfall, higher annual soil moisture levels and more annual vegetation cover than Sweden (electronic supplementary material, figure S3 and table S3). Accumulated heat and rainfall were more variable in space in Madagascar than in Sweden (as shown by more than double standard deviations), whereas spatial variation in soil moisture and vegetation cover was relatively similar between countries (as shown by less than double standard deviations; electronic supplementary material, figure S3 and table S3).

**Figure 1.**
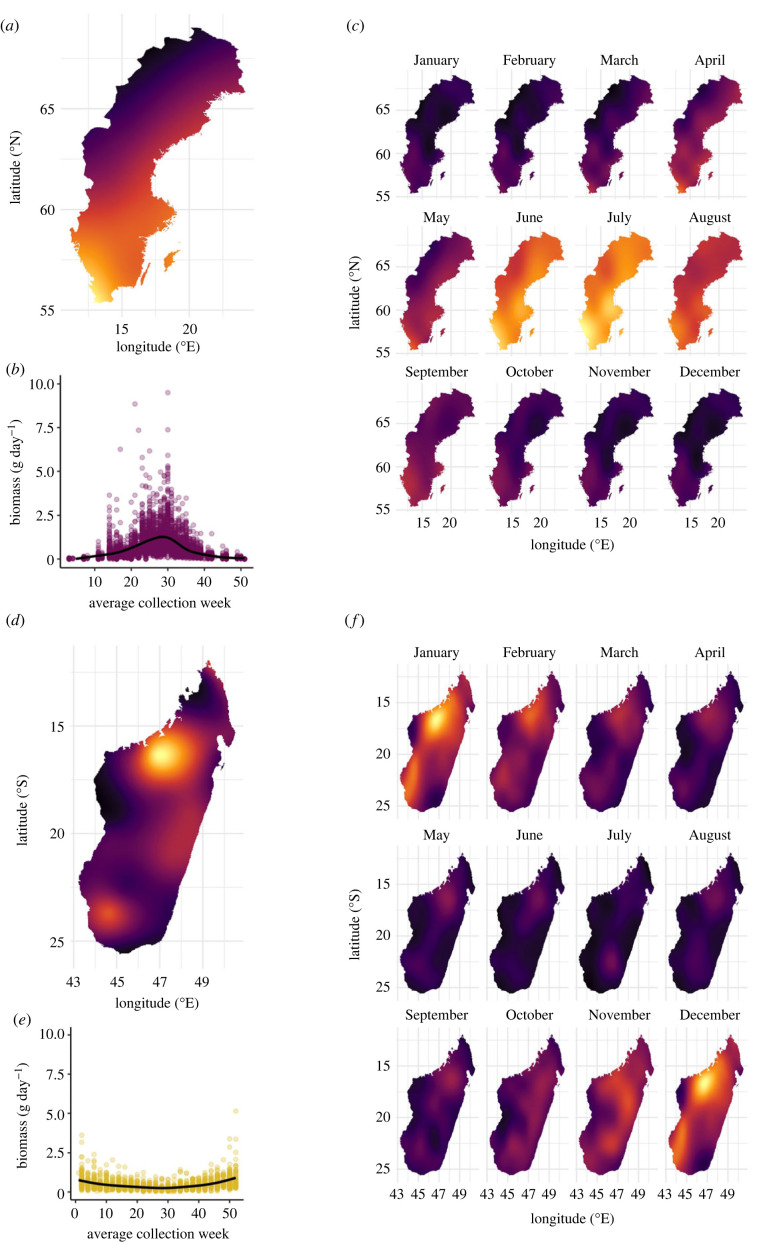
Patterns in the spatial and seasonal distribution of insect biomass in Sweden and Madagascar. The predicted spatial distribution of total insect biomass is shown in (*a*) for Sweden and (*d*) for Madagascar, with warmer colours indicating relatively higher values of flying insect biomass. Seasonal distribution is shown in (*b*) for Sweden and (*e*) for Madagascar. Here, purple (Sweden) and yellow (Madagascar) dots present the average biomass per day (biomass (g) in a sample/days of sample collection) for each sample, during the average week of sample collection (week number of average Julian day, where average Julian day = (Julian day start of collection+Julian day end of collection)/2. To visualize the seasonal pattern in active insect biomass, we fitted a smooth spline to the scatter. In Sweden, a few large biomass values occurred in spring, which are excluded from (*b*) to improve clarity of the overall pattern. For a figure including all biomass values, see electronic supplementary material, figure S5. (*c*,*f*) Spatiotemporal distribution of insect biomass throughout the year in Sweden and Madagascar respectively, with lighter (yellow) colours indicating higher values of flying insect biomass, and darker (purple) colours indicating lower values of flying insect biomass. Biomass predictions in (*a*,*c*,*d*,*f*) were based on generalized additive models. In (*a*,*d*), daily biomass was modelled as a function of latitude and longitude fitted as an interaction smooth. In (*c*,*f*), daily biomass was modelled as a function of latitude and longitude fitted as an interaction smooth, month fitted as a cyclic cubic regression spline, and the three-way interaction smooth between latitude, longitude and month fitted as a tensor product smooth with a cyclic cubic regression spline. For maps with raw data points, see electronic supplementary material, figure S5.

Flying insect biomass per trap per day was not detectably different between Sweden and Madagascar (figure 1; electronic supplementary material, figures S3*a* and S4*a*), either for the across-habitat comparison (Sweden: 0.46 ± 0.25 g, Madagascar: 0.45 ± 0.16 g), or when comparing Swedish forests (0.40 ± 0.18 g) with Malagasy forests (0.45 ± 0.16 g) (electronic supplementary material, table S4). Spatial variation in flying insect biomass was slightly higher across Sweden (s.d.: 0.25) than across Madagascar (s.d.: 0.16) (electronic supplementary material, figures S3*b* and S4*b*, and table S4). However, when focusing on forests alone, spatial variation in biomass was similar in Sweden and Madagascar (s.d.: 0.18 and 0.16 respectively; electronic supplementary material, figure S4*c* and table S4).

### (b) Drivers of the spatial distribution of flying insect biomass

**Figure 2.**
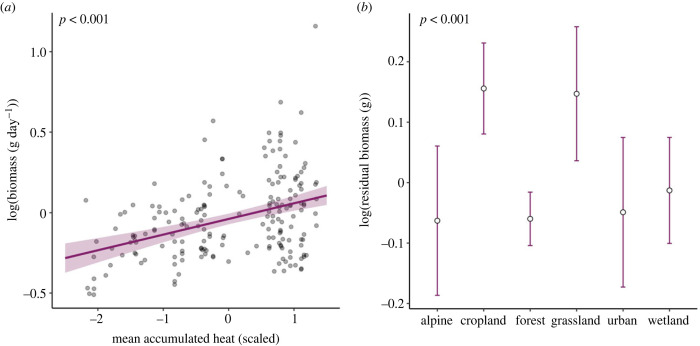
The effect of climate and habitat on total insect biomass in Sweden during 2019. Effect of (*a*) mean accumulated heat on the yearly total insect biomass, and (*b*) habitat types on the residual variation in biomass after accounting for the effect of accumulated heat. In (*a*), the solid trendline presents the significant relationship predicted by the model. In (*b*), circles represent estimated means with confidence intervals in purple for each habitat type as based on model predictions. The *p*-values of predictors are presented in the upper left corners. For model output related to this figure, including *p*-values, *F*-values and degrees of freedom, see electronic supplementary material, table S5a.

Flying insect biomass per trap per day in Sweden increased with mean annual accumulated heat (figure 2*a*, *F*_1,189_ = 32.49, *p* < 0.001), while snow depth, rainfall, soil moisture and the interaction between accumulated heat and rainfall were not retained in the model (electronic supplementary material, table S2). Differences in habitat type explained part of the residual variation in flying insect biomass (*F*_5,184_ = 6.42, *p* < 0.001), with the highest biomass of flying insects observed in croplands and grasslands (figure 2*b*; electronic supplementary material, table S5*a*). Vegetation cover had no significant effect on residual variation in flying insect biomass (electronic supplementary material, table S5*a*).

In Madagascar, mean annual accumulated heat had no detectable effect on flying insect biomass per trap per day (electronic supplementary material, figure S6*a*, *F*_1,48_ = 0.89, *p* = 0.35). Rainfall, soil moisture and the interaction between accumulated heat and rainfall were not retained in the model (electronic supplementary material, table S2). Residual variation in the spatial distribution of flying insect biomass was not detectably affected by habitat type, vegetation cover, forest cover or canopy cover (electronic supplementary material, figure S6*b–d* and table S5*b*).

### (c) Temporal variation in climate and flying insect biomass in Sweden and Madagascar

Temporal variation in accumulated heat and vegetation cover was higher in the temperate zone, while temporal variation in rainfall and soil moisture was higher in the tropics (electronic supplementary material, figure S3 and table S3).

In Sweden, flying insect biomass varied significantly more throughout the year than it did in Madagascar, both across habitats (standard deviation per trap: Sweden 0.65 ± 0.21 g Madagascar 0.30 ± 0.16 g, *p* < 0.001; figure 1; electronic supplementary material, figure S3*b* and table S4) and between Swedish and Malagasy forests (standard deviation per trap Swedish forests: 0.58 ± 0.12 g, Malagasy forests 0.30 ± 0.16 g; electronic supplementary material, figure S4*d* and table S4).

### (d) Drivers of the temporal distribution of flying insect biomass

**Figure 3.**
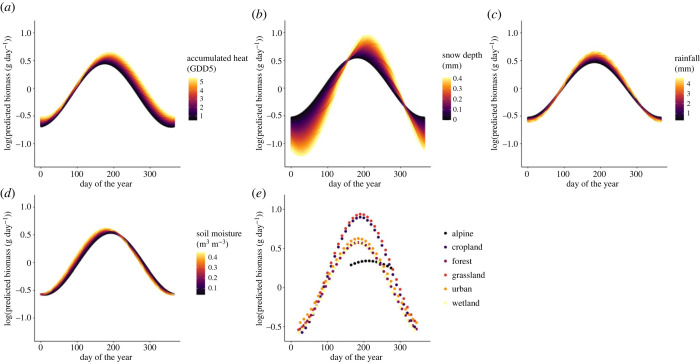
The predicted effects of climatic and landscape variables on insect seasonality in Sweden. Predicted seasonal trends of insect biomass in response to (*a*) yearly mean accumulated heat, (*b*) yearly mean snow depth, (*c*) yearly mean rainfall, (*d*) yearly mean soil moisture and (*e*) habitat type. Shown are marginal effects, that is, the impact of the focal factor with all other factors fixed at their mean level. Colour gradients represent the range of values of each of the environmental predictors, with lighter colours indicating higher values. The *x*-axis displays the day of the year, where day 1 is 1 January. For each of the habitat types (*e*), we only show predictions for the time-period during which sampling took place in a particular habitat. For model output related to this figure, including *p*-values, *χ*^2^-values and degrees of freedom, see electronic supplementary material, table S6*a*.

In Sweden, seasonality of insects was significantly influenced by accumulated heat, snow depth, rainfall, soil moisture and habitat type (figure 3*a–d*; electronic supplementary material, table S6*a*), while vegetation cover was not retained in this model (electronic supplementary material, table S2). Locations with a higher yearly mean of accumulated heat had a higher peak in flying insect biomass during the growing season. Such locations also showed a later decrease in biomass at the end of the growing season, and sustained slightly higher biomass during the off-season (figure 3*a*). Locations with higher mean annual snow depth had lower biomass during winter, and started to accumulate biomass later during the growing season (figure 3*b*). Interestingly, locations with more snow during the winter were also characterized by higher peak biomass during the late summer (figure 3*b*). Locations with more annual rainfall had higher flying insect biomass during summer, but lower biomass during winter (figure 3*c*). Locations with higher soil moisture tended to accumulate biomass slightly earlier than drier locations (figure 3*d*). Seasonality also differed among habitats, where grasslands and croplands had the highest peak biomass, while alpine areas had the lowest (figure 3*e*). Moreover, biomass in alpine areas peaked slightly later during the growing season than in any other habitat (figure 3*e*).

**Figure 4.**
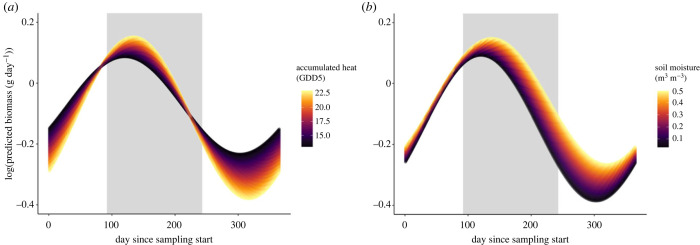
The predicted effects of climatic and landscape variables on insect seasonality in Madagascar. Predicted seasonal trends of insect biomass in response to (*a*) accumulated heat and (*b*) soil moisture. Shown are marginal effects, that is, the impact of the focal factor with all other factors fixed at their mean level. Colour gradients represent the range of values of each of the environmental predictors, with lighter colours indicating higher values. The *x*-axis displays the day number since the start of sample collection, where day 1 is 1 August. The grey area in the plot indicates the rainy season (November to May). For model output related to this figure, including *p*-values, *χ*^2^-values and degrees of freedom, see electronic supplementary material, table S6*b*.

In Madagascar, the seasonality of insects was significantly influenced by accumulated heat and soil moisture (figure 4, electronic supplementary material, table S6*b*), while rainfall, vegetation cover and habitat type were not retained as predictors (electronic supplementary material, table S2). Locations with a higher annual mean of accumulated heat had higher flying insect biomass during the rainy season, but less biomass during the dry season (figure 4*a*). Locations with higher annual soil moisture had higher flying insect biomass throughout the year compared to drier locations (figure 4*b*). The flying insect biomass of both wet and dry locations peaked during the start of the rainy season, but wetter locations sustained a longer period of peak insect activity (figure 4*b*).

### (e) Drivers of short-term deviations in biomass

In Sweden, residual variation in the seasonal distribution of biomass could be explained by weekly fluctuations in weather. Warmer, moister periods had higher flying insect biomass and periods with more rain led to lower flying insect biomass (electronic supplementary material, figure S7 and table S7*a*).

In Madagascar, weekly fluctuations in soil moisture left the only statistically detectable imprint on residual variation in the seasonal distribution of biomass. Here, weeks with higher soil moisture had higher flying insect biomass than expected from seasonality trends alone (electronic supplementary material, figure S8*c* and table S7*b*). Weeks with higher accumulated heat tended to show higher relative biomass, but this effect was not statistically significant (electronic supplementary material, figure S8*a* and table S7*b*). Fluctuations in rainfall explained no significant part of the residual variation in flying insect biomass (electronic supplementary material, figure S8*b* and table S7*b*).

## 4. Discussion

**Figure 5.**
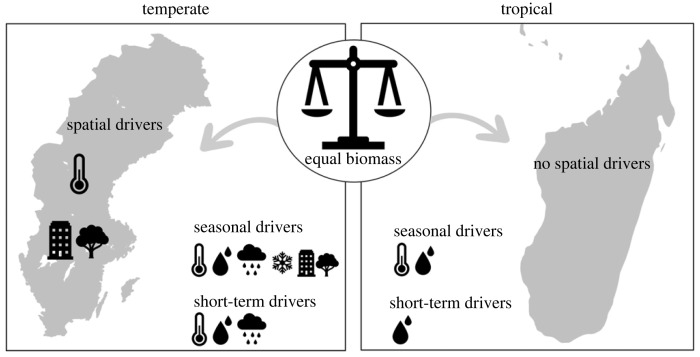
Visual overview of the main findings of this study. The average weight of insect biomass across the year was similar for the temperate region (Sweden, left) and the tropical region (Madagascar, right). In the temperate region, *spatial* variation in insect biomass was driven by accumulated heat and habitat type, whereas in the tropical region, none of the candidate drivers measured had a detectable impact on biomass. In the temperate region, *seasonal* variation in insect biomass was driven by accumulated heat, soil moisture, rainfall, snow depth and habitat type, whereas in the tropical region, seasonal variation was driven by accumulated heat and soil moisture. In the temperate region, *short-term* variation in insect biomass was driven by accumulated heat, soil moisture and rainfall, whereas in the tropical region, short-term variation was driven by soil moisture.

By intensively sampling the insect fauna across a tropical and a temperate country, we were able to reveal general as well as region-specific patterns and drivers of flying insect biomass distribution and seasonality (figure 5). While the amount of flying insect biomass is often assumed to be higher in tropical than temperate regions, we found a surprising convergence in daily averages across climatic zones. Nonetheless, temporal variation in biomass was higher for the temperate than for the tropical zone. In terms of the spatial distribution of flying insect biomass, the drivers proved different for the temperate and tropical zones, whereas in terms of insect seasonality, the drivers were partly similar. In particular, accumulated heat and water availability affected insect seasonality in both climate zones. Besides seasonal patterns, weeks with higher soil moisture showed higher flying insect biomass in both the temperate and tropical zone, while warmer weeks with less rain explained increases in biomass only in the temperate zone. While our study provides seminal insights into the patterns and drivers of the spatial and temporal distribution of insect faunas in a tropical and temperate region, future studies across multiple countries are needed to confirm the generality of our findings across a wider range of latitudes.

### (a) Local biomass is similar in Sweden and Madagascar

While a recent meta-analysis suggests the biomass of soil arthropods to be higher in tropical forests than temperate forests or grasslands [7], we found no difference in the biomass of flying insects between Sweden and Madagascar. This observation fills a key knowledge gap left open by Rosenberg *et al*. [7], as previous studies of aboveground arthropods proved too scarce to compare biomasses across biomes. Indeed, previous studies of flying insect biomass have primarily been focused on the temperate zone [6,11,12]. As these earlier studies sampled insects only during the growing season, they tend to overestimate year-round biomass, which precludes reliable comparisons with the tropical zone. Our year-round sampling campaign allows a direct comparison of total flying insect biomass between a temperate and tropical country. However, since our sampling in Madagascar was explicitly focused on forests, whereas sampling in Sweden included other habitat types too, we should pay special attention to the comparisons among forests alone. This comparison showed similar flying insect biomasses per day in Swedish and Malagasy forests. Notably, equal amounts of year-round biomass in the temperate and tropical region do not translate into similarities in species richness, diversity or abundances, as similar amounts of biomass may represent vastly different numbers of species or individuals. Moreover, Masteller [61] observed that insects were typically smaller in a tropical (Puerto Rico) than a temperate (Pennsylvania) site. Thus, any given mass of insect biomass collected in the tropics could contain more insects than the same mass collected from a temperate region.

In terms of spatial variation in flying insect biomass, we found slightly more variation in Sweden than in Madagascar. This general contrast can be attributed to a larger variety of habitat types being sampled in Sweden, since no differences emerged among Swedish and Malagasy forests. To gain reliable estimates of flying insect biomass across biomes without biases towards seasons, taxa or habitat types [7], extensive sampling campaigns such as ours are urgently needed. Future projects should aim at year-round sampling, while covering biomes across the globe.

### (b) Drivers of spatial variation in biomass differ between Sweden and Madagascar

In Sweden, local flying insect biomass increased with accumulated heat, and was higher in grasslands and croplands than in forests, wetlands, alpine or urban areas. In Madagascar, the spatial distribution of flying insect biomass was unrelated to any of the climate or landscape predictors measured. This difference between zones seems reflective of the additional climatic constraints on insect physiology imposed by temperate conditions. In the temperate zone, low temperatures are likely to limit insect survival and activity [62] (electronic supplementary material, figure S3), and other studies in the temperate zone have reported equally strong impacts of temperature on insect biomass [6,11,12,63]. By comparison, tropical temperatures are typically high enough to sustain insect activities year-round [5] (electronic supplementary material, figure S3). Given that temperature affected the spatial distribution of temperate but not tropical insect biomass, one could predict that insect biomass in the temperate zone will be more sensitive to climate change compared with insect biomass in the tropics. Nevertheless, tropical insects tend to have narrower windows of thermal tolerance than their temperate counterparts [16]. Thus, tropical insects may be disproportionally sensitive to changes in temperature, and future increases in temperature may thus prove harmful to both temperate and tropical insects. As a further alternative, the spatial distribution of flying insect biomass in the tropics may also be determined by biotic rather than abiotic conditions [64]—a hypothesis worth further exploration.

With climatic effects accounted for, the amount of flying insect biomass differed among habitat types in Sweden. We found flying insect biomass to be highest in open, semi-natural and agricultural landscapes (grasslands and croplands), but lower in forests, urban areas, wetlands and alpine areas—a pattern that is fully or partially consistent with other findings from the temperate zone [11,12]. Grasslands and croplands can potentially sustain higher flying insect biomass owing to high plant productivity [65], with added effects of fertilization in croplands [66]. Alternatively, the open landscape of grasslands and croplands might promote insect movement, which could increase local trap catches [67]. Insect communities in forests are more vertically stratified, from the forest floor to the canopy [28], and trap catches may thus underestimate the overall biomass [68,69]. In the tropics, we found no differences in flying insect biomass between dry forest and rainforest, and no effect of vegetation cover, after accounting for climatic conditions. Hence, even though species richness and diversity are generally assumed to be higher in rainforests than dry forests [70], we saw no such pattern in total biomass. All in all, our results illustrate that human land use changes can cause shifts in the amount of insect biomass. Based on our findings, increased urbanization in the future could lead to decreases in local insect biomass, while agricultural expansion may instead increase local insect biomass. Notably, increases in insect biomass due to land-use changes can still go hand-in-hand with impoverishment of insect species richness and diversity [11].

### (c) Drivers of insect seasonality partly converge between countries

Consistent with earlier studies [71,72], we found seasonal fluctuations of flying insect biomass to be more pronounced in the temperate than tropical zone. Nonetheless, the underlying drivers appeared partly similar. In both Sweden and Madagascar, the seasonality of flying insect biomass differed between warmer and colder sites as well as between wetter and drier sites. In Sweden, seasonality also differed between sites with higher or lower snow depth in winter, and among habitat types. In the temperate zone, biomass during the growing season was highest in the warmest locations. Furthermore, flying insect biomass declined later in warmer locations than in colder locations. Both patterns are supported by previous findings regarding phenological responses of insects to temperature [34,35]. In the tropical zone, the effects of accumulated heat on insect seasonality were linked to the timing of the rainy and dry seasons. Warmer locations showed higher biomass during the rainy season, but lower biomass during the dry season than did cooler locations. Thus, while heat promotes insect activity during rainy periods, heat can accelerate desiccation, mortality or inactivity of insects during the dry season [5]. Based on our findings, we predict that insect seasonality could be strongly affected by rises in temperature [73]. In the temperate region, warming may increase the peak insect biomass during the growing season, and the end of the growing season may be extended. Yet, once thermal maxima are reached, this positive effect may well reverse [74]. As tropical insects are adapted to relatively stable temperature regimes, their thermal tolerances may be lower compared with those of temperate insects [16,17]. Hence, even slightly elevated local temperatures may exceed the thermal maxima of tropical insects, and could have negative effects on peak insect biomass—especially so for dryer regions [75]. For Sweden and other temperate regions, insects are moving northwards with climate warming [76,77]. In the tropics, opportunities for large-scale migration to cooler regions are more limited—and even more so for insects in insular regions, like Madagascar.

Water availability affected insect seasonality in both the temperate and tropical zones. In Sweden, rainy locations sustained more biomass during the growing season, but less during the off-season compared with less rainy locations. This pattern suggests accentuated benefits of precipitation during periods of limited water availability [78]. During the off-season, more rain may limit flying time and increase mortality [21,22]. Wet locations also showed an earlier start of the growing season, and a higher peak biomass during the growing season, than did drier locations. Soil moisture may thus be particularly important during the start of the growing season, when overwintering insects in the soil emerge, hatch or eclose [79,80]. Additionally, soil moisture might promote plant growth and vigour at the start of the growing season [81], favouring insects dependent on plants for food or shelter. In the tropics, soil moisture also had a strong effect on insect seasonality. Dry locations had lower flying insect biomass throughout the year, and this difference was most pronounced during the first half of the dry season. Dry and wet locations both peaked in flying insect biomass during the rainy season, but dry locations had a shorter period of insect activity and lower overall biomass. Tropical sites with higher water availability can thus sustain higher abundances of insects for longer periods of time [43]. As climate change is likely to cause prolonged droughts and more extreme episodes of rainfall in many regions [73], our results suggest that the timing and the amount of insect biomass are likely to change across zones.

In Sweden, snow depth emerged as another important driver of latitudinal differences in insect seasonality. Locations with more snow had lower biomass during the off-season, but higher biomass during the growing season. Deeper snow could increase soil moisture during spring and summer, and insulate diapausing insects in winter [36–38], with both mechanisms contributing to higher summer-time insect abundances. Our findings suggest that climate change could alter the seasonality and abundance of insects in temperate regions via changes in snow regimes. A reduction in snow cover is predicted under scenarios of climate change and has been observed empirically in Nordic countries [82]. This may increase insect mortality due to freezing in winter and lower water availability in summer, resulting in lower insect biomass during the growing season [36,37].

### (d) Abiotic factors explain short-term variation in biomass

Short-term deviations from the season-specific expected biomass in Sweden were attributable to week-to-week fluctuations in accumulated heat, rainfall and soil moisture. In Madagascar, only short-term fluctuations in soil moisture contributed to temporal variation in biomass. In line with our *a priori* expectations (electronic supplementary material, table S1), warmer periods with wetter soils showed more biomass than expected under Swedish conditions, while periods with more rainfall had less biomass than expected. In Madagascar, periods with wetter soils had more biomass than expected. While warmer periods tended to have more biomass, this effect was not significant. The weak or absent effect of temperature on short-term fluctuations in flying insect biomass in Madagascar matches the notion of lower climatic constraints on insect activity in the tropics. In Madagascar, temperatures are generally warm (electronic supplementary material, figure S3*c*) and relatively stable year-round (electronic supplementary material, figure S3*d*). By comparison, water availability emerges as a likely limiting factor for insect activity, especially during the dry season. This is supported by our finding of seasonality patterns—where the peak of flying insect biomass was higher for sites with higher soil moisture—and it is consistent with previous findings of higher insect biomass with moister conditions in the tropics [24,75]. Then again, weekly rainfall had no detectable effect on flying insect biomass in Madagascar, possibly owing to some conflicting impacts of rain on insect activity: on the one hand, more rain means more available water, which is expected to promote insect activity [83], but on the other hand, insect flight could be limited during periods of rainfall [46]. Given current predictions of massive changes in precipitation patterns with climate change [73], future studies should explore the trade-offs between positive and negative effects of precipitation on insect performance and seasonality.

## 5. Conclusion

Our study identified several key patterns and drivers of the spatial distribution and seasonality of temperate and tropical insect faunas (figure 5). Our findings have major implications for the impacts of climate change and land-use on the spatial distribution of insect biomass in temperate and tropical regions. While temperature affected the spatial distribution of insect biomass only in the temperate region, and not in the tropics, insects in both regions can still be affected by rising temperatures in the coming decades. Increased droughts and more extreme periods of rainfall—as predicted under scenarios of climate change—may negatively affect the total biomass of tropical as well as temperate insects. The timing and amplitude of seasonality curves are also likely to shift during the coming decades in response to climate change [84]. The direction of this shift is hard to predict, since shifting climate variables may have opposing effects on seasonality: based on our findings for the temperate zone, the insect growing season may be *extended* and peak *higher* in response to rising temperatures, while at the same time, snow melt may cause the growing season of insects to shift *earlier* and peak *lower*. Moreover, if rising temperatures exceed the thermal tolerances of temperate insects, impacts on seasonality could reverse. Altered seasonality could have consequences for the temporal availability of functions and food provided by insects. More generally though, such changes could lead to phenological mismatches across trophic levels, with implications for the functioning of the entire food web [85]. As a next step, future studies could aim to describe the cues that determine the emergence and decline, as well as peak abundance, of various taxa throughout seasons across climate zones. Ideally, such studies would focus on multiple taxa from different trophic levels, comparing their phenological changes in response to climate, and identifying or predicting the emergence of potential mismatches [85,86]. Such knowledge will enable us to determine whether the probability for mismatches differs across climatic zones. Uncovering the patterns and drivers of insect distribution and seasonality across geographical regions will allow us to understand the seasonal availability of insects to provide food and ecosystem functions, and inform predictions of future insect biomass declines across the globe.

## Data Availability

Data are archived in the Dryad repository [87]. Supplementary material is available online [88].
